# Elevated temperature undermines the efficacy of *Streptococcus pyogenes* dCas9-based CRISPRi systems

**DOI:** 10.1128/msphere.00379-26

**Published:** 2026-08-19

**Authors:** Lili M. Kim, Byoung-Mo Koo, Carol A. Gross, Horia Todor

**Affiliations:** 1 Department of Microbiology and Immunology, University of California, San Francisco 8785, San Francisco, California, USA; 2 Department of Cell and Tissue Biology, University of California, San Francisco 8785, San Francisco, California, USA; 3 California Institute of Quantitative Biology, University of California, San Francisco 8785, San Francisco, California, USA; University of Nebraska Medical Center College of Medicine, Omaha, Nebraska, USA

**Keywords:** CRISPRi, heat shock, *Bacillus subtilis*

## Abstract

Targeted gene repression using CRISPRi has become a foundational technique for microbiology. The effector protein in most CRISPRi systems is a catalytically inactivated version of Cas9, an RNA-guided DNA endonuclease from *Streptococcus pyogenes* (SpCas9). The application of SpCas9-based CRISPRi systems in increasingly ambitious experiments in diverse bacteria raises important questions about the effects of growth temperature on CRISPRi efficacy, particularly because SpCas9 loses cutting efficacy at elevated temperatures. Here, we test the effect of temperature on SpCas9*-*based CRISPRi knockdown efficacy by performing a series of genome-wide screens using a well-characterized CRISPRi system in the model firmicute *Bacillus subtilis*. We observe very little repression at high (45°C) growth temperatures and confirm this observation using single cell measurements of GFP fluorescence in CRISPRi strains grown at different temperatures. Our data suggest that *Streptococcus pyogenes-*based CRISPRi systems are ill-suited for thermophiles and should not be used for elevated-temperature or heat shock experiments in mesophilic bacteria.

**IMPORTANCE:**

CRISPRi is a common technique for inducible gene depletion in bacteria and has been used for many different types of experiments in increasingly diverse bacteria. We identify increased temperatures as an important limitation of the most common CRISPRi effector (*Streptococcus pyogenes* dCas9). This finding both affects experimental design and informs the kinds of bacterial species amenable to manipulation by *S. pyogenes*-based CRISPRi systems.

## OBSERVATION

CRISPRi systems have revolutionized the study of essential genes in bacteria by facilitating inducible and titratable control of their expression (1, 2). Bacterial CRISPRi systems typically co-opt class 2, type II-A CRISPR-Cas systems (3, 4), which are natively used by bacteria to protect against phages and other mobile genetic elements (5, 6). An inactivated nuclease effector protein (e.g., dCas9) is targeted to a specific DNA sequence by a complementary RNA, usually a single guide RNA (sgRNA) (3). The dCas9-sgRNA ribonucleoprotein (RNP) complex binds in a two-step process: first, dCas9 recognizes a short DNA sequence (e.g., NGG for Sp*-*dCas9) called the protospacer adjacent motif (PAM); second, dCas9 progressively unwinds the DNA to interrogate sequence complementarity, ultimately forming a DNA:sgRNA R-loop that stabilizes the RNP (7). The fully bound dCas9 RNP sterically blocks RNA polymerase, leading to transcriptional inactivation of the targeted gene (3). dCas9 from *Streptococcus pyogenes* (Sp-dCas9) is by far the most commonly used effector, in both eukaryotic and bacterial engineered systems, and its performance depends on sgRNA sequence, expression system design, and other factors (8–10). *S. pyogenes* grows optimally at 37°C, and no growth is observed at 45°C (11). Previous work has demonstrated that SpCas9 has no nuclease activity *in vivo* at 42°C or above (12) or *in vitro* at 45°C or above (13) and becomes denatured at ~45°C (14), while Sp-dCas9 targeted to the origin of replication of *Escherichia coli* can block initiation at 37°C but not at 42°C (15). These data and the increasing use of Sp-dCas9-based CRISPRi systems in diverse bacteria with different optimal growth temperatures (16) prompted us to test whether CRISPRi repression efficacy is similarly affected by bacterial growth temperatures.

### High temperature, but not media or low temperature, affects CRISPRi activity

**Fig 1:**
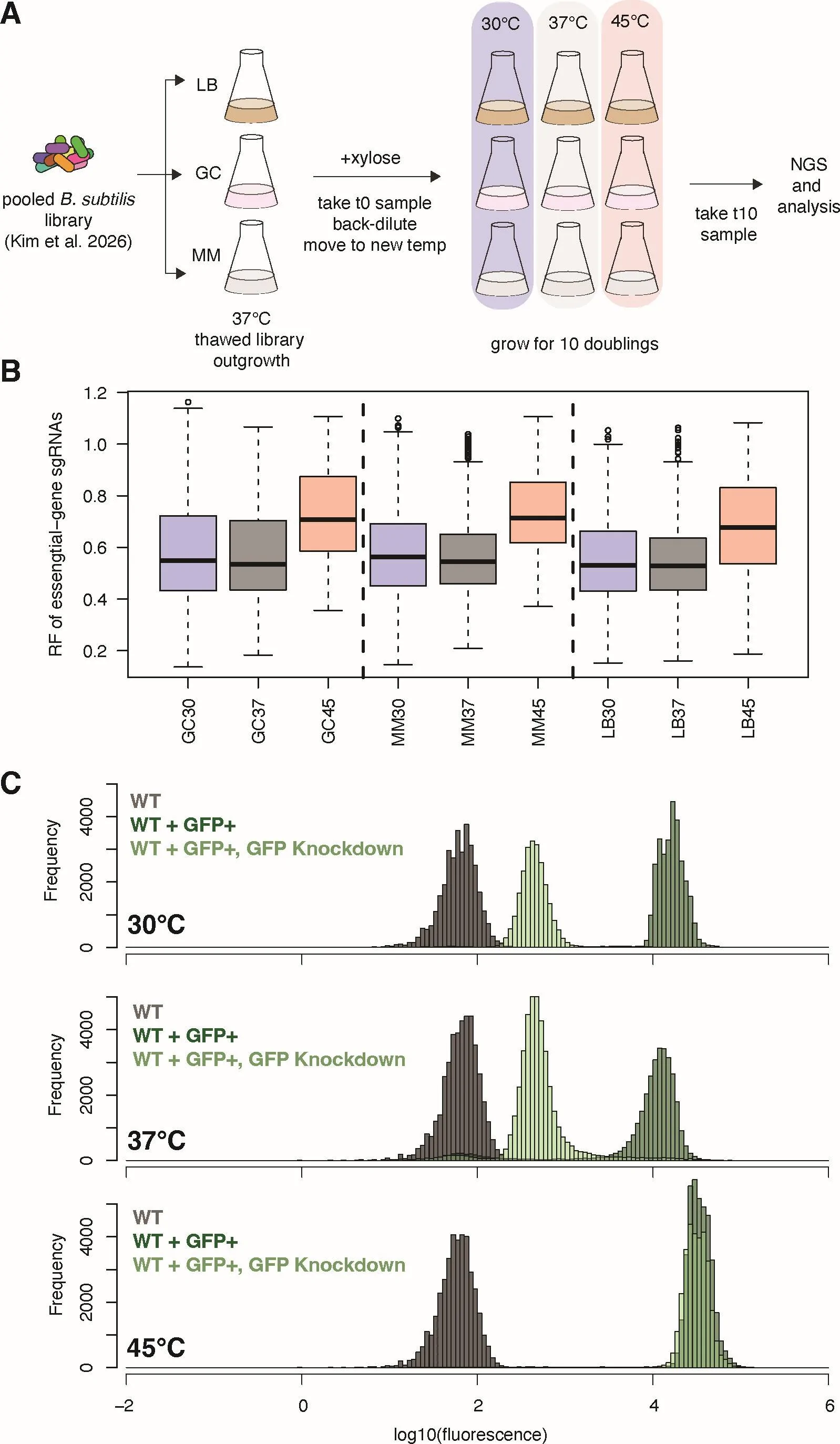
High temperature (45°C), but not different media types, affects CRISPRi knockdown efficacy. (**A**) Schematic of experiment and/or analysis. (**B**) Boxplot of RF values for fully complementary sgRNAs targeting essential genes in *B. subtilis* grown in different media (LB, minimal media [MM], and glucose complete [GC]) and in temperature (30°C, 37°C, and 45°C) conditions. High temperature is highlighted in red, and low temperature is highlighted in blue. Median RF for non-essential genes was not affected by media or temperature (Fig. S2). (**C**) Histograms of GFP fluorescence measured by FACS (methods) in three strains (gray: WT, dark green: WT + *gfp*, light green: WT + *gfp* + *CRISPRi*) at three temperatures (30°C, 37°C, and 45°C), showing efficacious knockdown at 30°C and 37°C but not at 45°C.

To test the effect of media and temperature on CRISPRi efficacy, we performed a pooled CRISPRi growth screen in *Bacillus subtilis*, a gram-positive model organism that grows well at a range of temperatures (30°C–45°C) and media (Fig. S1). We used a previously described CRISPRi system that has been well characterized across a range of different modalities (CRISPRi [17], mismatch-CRISPRi [18], and double-CRISPRi [19]) and a previously described sgRNA library (20) targeting every gene with two fully complementary sgRNAs (plus additional lower efficacy sgRNAs for essential genes). We grew the library at 30°C, 37°C, and 45°C in three different media conditions (LB, minimal media [MM], and glucose complete) for 10 generations and assessed the effect of temperature on the depletion of sgRNAs targeting essential genes using next-generation sequencing (Fig. 1A; Table S1). Doubling times varied between 14 min (LB at 45°C) and 93 min (MM at 30°C): for each media type, growth was slowest at 30°C and fastest at 45°C (Fig. S1).

To quantify the effects of media and temperature on the efficacy of the CRISPRi system, we focused on the growth, as quantified by relative fitness (RF), of strains targeting essential genes. We reasoned that while many strains targeting non-essential genes (e.g., auxotrophs) and specific essential genes (e.g., those encoding ribosomal proteins) may have different growth phenotypes in different media or different temperatures irrespective of CRISPRi efficacy, essential genes as a whole are likely similarly essential in different media and/or temperatures, an assumption supported by the overwhelming conservation of essential genes in evolutionarily diverged bacteria with vastly different growth requirements (21). Our CRISPRi library (20) targeted each of the 307 essential or near-essential (22) genes of *B. subtilis* with two fully complementary sgRNAs. sgRNAs meeting QC standards (20) exhibited similar median RF in all media at 30°C and 37°C (Fig. 1B) but a significantly higher median RF in all media at 45°C (*t*-test *P* values between 2.23 × 10^−25^ and 2.78 × 10^−51^), indicating that while media composition does not generally affect CRISPRi efficacy, high temperatures (e.g., 45°C) drastically decrease knockdown efficacy.

To verify that this result was not due to a general effect of temperature on gene essentiality, we used flow cytometry to test the ability of our CRISPRi system to repress constitutively expressed *gfp*, a synthetic gene, at various temperatures. In the absence of CRISPRi repression, GFP accumulated to similar levels at 30°C, 37°C, and 45°C (Fig. 1C). Consistent with previous results, our CRISPRi system significantly repressed *gfp* expression (~44-fold) at 37°C. Repression was similar at 30°C (~47-fold), but almost no repression was observed at 45°C (~2.7-fold), suggesting that growth at 45°C drastically reduces the efficacy of CRISPRi knockdown.

### Four temperature-sensitive knockdown strains

The low CRISPRi efficacy at 45°C affected sgRNAs targeting genes involved in different processes approximately equally (Fig. S3; [23]), making it difficult to ascertain the biological effects of specific gene knockdown on growth at 45°C. However, knockdown strains targeting four genes consistently (i.e., in at least 2/3 media tested) grew significantly (ΔRF >0.2) worse at 45°C than at 37°C: *divIB*, *thyA*, *ctsR*, and *yoaA*. DivIB is known to be required for cell division at high temperature (24), and ThyA was previously found to be essential at high temperature due to the thermolability of its paralog (25). CtsR (repressor of the class III heat shock genes [26]) and YoaO (a DUF4944 protein) have not been previously found to be important for growth at elevated temperatures and may represent novel players in this process.

### sgRNA sequence determinants of temperature sensitivity

**Fig 2:**
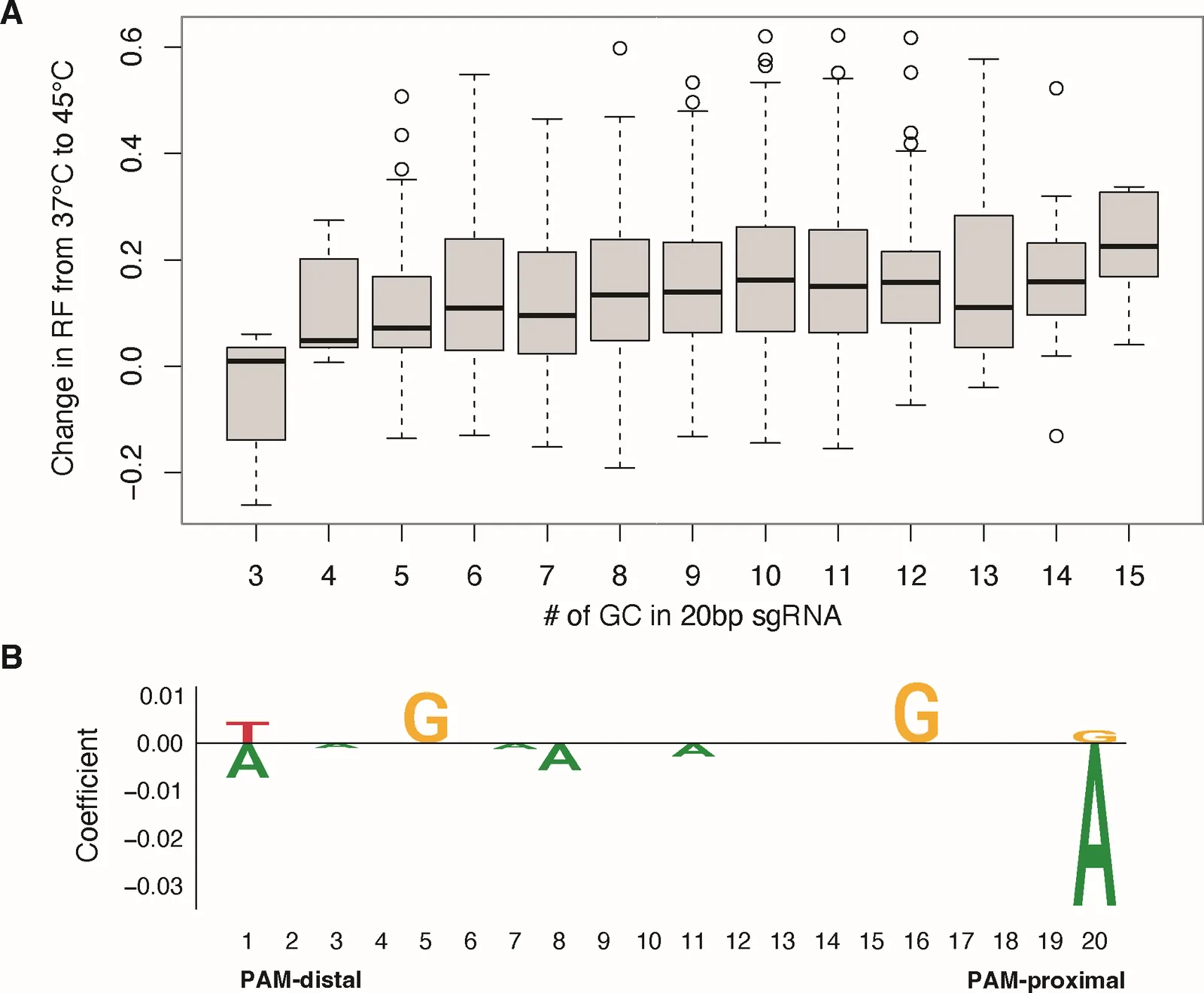
Sequence determinants of CRISPRi efficacy loss at high temperature. (**A**) Boxplot showing the change in RF values for fully complementary sgRNAs targeting essential genes between 37°C and 45°C by the number of G/C nucleotides in the sgRNA. More GC-rich sgRNAs lose more efficacy between 37°C and 45°C than lower GC sgRNAs. (**B**) Sequence logo of L1 regularized linear regression coefficients between the one-hot encoded nucleotides in the sgRNA sequence and the loss of efficacy of the sgRNA at high temperature, as measured by the change in RF between 37°C and 45°C. Positive coefficients represent sgRNA features associated with a greater loss of efficacy at 45°C (i.e., higher change in RF between 37°C and 45°C); negative coefficients represent sgRNA features associated with a decreased loss of efficacy at 45°C (i.e., lower change in RF between 37°C and 45°C).

Not all sgRNAs exhibited similar decreases in efficacy at 45°C. Since the melting temperature of an RNA:DNA duplex is affected by its sequence composition, we first analyzed the effect of sgRNA GC content on the change in knockdown efficacy. Counterintuitively, we found that fully complementary sgRNAs targeting essential genes with a higher than average GC content (GC ≥45%) lost more efficacy at 45°C than sgRNAs with lower than average GC content (mean change in RF from 37°C to 45°C; low GC = 0.134, high GC = 0.165; *t*-test *P* = 5.77×10^−6^; Fig. 2A). This suggests that melting temperature is not the major determinant of CRISPRi efficacy at high temperature.

To determine if specific aspects of the sgRNA sequence could make an sgRNA more or less sensitive to temperature, we fitted a linear model on the one-hot encoded primary sequence of the sgRNAs to predict their change in RF when moving from 37°C to 45°C. Consistent with previous work to predict the sequence determinants of sgRNA knockdown efficacy (8, 10), we used L1 regularization and 10-fold cross-validation, which pushes coefficients to zero to avoid overfitting. The coefficients of this model, visualized as a sequence logo (Fig. 2B), highlighted two major aspects of sgRNA sequence that contribute to the sensitivity of individual sgRNAs to elevated temperature. First, having an “A” at the PAM-proximal nucleotide appears to partially mitigate efficacy loss at 45°C. It is unclear why this may be the case, but it does not appear to be caused by those sgRNAs being stronger overall; sgRNAs with a PAM-proximal “G” also have high knockdown efficacy (8, 10) but are less efficacious at 45°C than others (Fig. 2B). Second, sgRNAs with a PAM-distal “A” were less affected by transitioning from 37°C to 45°C and those with a PAM-distal “T” were more affected. Since the PAM-distal position is also the transcription start site of the sgRNA, this is likely due to *B. subtilis-*specific differential changes in transcriptional efficacy for different initiating nucleotides, analogous to those observed at 37°C (8).

## SUMMARY AND PERSPECTIVE

A great advantage of CRISPRi tools is their applicability in biological systems evolutionarily distant from their native host. For example, SpCas9-based CRISPRi systems have been used in other firmicutes, proteobacteria (e.g., *E. coli*), and eukaryotes. Our work has uncovered a limitation in the use of SpCas9-based CRISPRi systems: repression is lost at elevated temperatures (e.g., 45°C), making this CRISPRi system unsuitable for heat-shock experiments and for bacteria that are routinely cultured at temperatures >37°C. This result is consistent with previous *in vivo* and *in vitro* work demonstrating SpCas9 thermolability and denaturation at elevated temperatures (12–15), and expands those observations to its use in CRISPRi. While specific sgRNA sequence characteristics can partially mitigate the loss of efficacy, effectively applying CRISPRi at elevated temperatures likely requires the use of thermophilic Cas9 proteins, such as those from *Streptococcus thermophilus*.

## METHODS

### General strain manipulations and procedures

Unless otherwise noted, all growth assays for *B. subtilis* were done in LB medium. LB, glucose complete, and minimal media were prepared as previously described (22).

### Whole-genome CRISPRi library experiments and sequencing

A previously described *B. subtilis* CRISPRi library was used (20) for all experiments.

Library aliquots were thawed, diluted to an OD600 of 0.01, and grown in the appropriate media at 37°C to an OD600 of 0.10. From here, a *t*_0_ sample was taken, and the culture was back-diluted to an OD600 of 0.01 in the appropriate media pre-warmed to 30°C, 37°C, or 45°C with 1% xylose to induce dCas9 expression. After approximately five doublings, the culture was back diluted 1:10 into fresh media + 1% xylose; after another five doublings, the culture was pelleted and frozen. OD600 was measured at each back-dilution and before collection via the Synergy H4 plate reader (BioTek) calibrated to OD600 spectrophotometer readings. Genomic DNA was extracted from samples with the DNeasy Blood and Tissue Kit (QIAGEN), and libraries were prepared, pooled, and sequenced using Illumina NovaSeq as previously described (18, 20). Experiments were performed in triplicate.

### Relative fitness analysis

Relative fitness values were calculated from sequence counts as previously described (18, 20). Briefly, we calculate the relative fitness *F*(*x*) as


$$F\left(x\right)=\;\frac{log2\frac{r_{wt}\left(t_{0}\right)\;\times \;r_{x}\left(t_{10}\right)}{r_{wt}\left(t_{10}\right)\;\times \;r_{x}\left(t_{0}\right)}}{g_{wt}}+1,$$

where *r_x_*(*t*_0_) is the fraction of strain *X* in the population at time *i*, and *g*_wt_ is the number of doublings of wild-type growth in the experiment. *g*_wt_ is calculated from OD measurements. For this screen, *r*_wt_(*t_i_*) is calculated as the median value of non-essential guides. Experimental samples with a median NE gene knockdown |L2FC| >0.6 were discarded. Additionally, replicates with Pearson’s *r* < 0.8 with the other two replicates of the same condition were discarded. In contrast to previous studies, RF values were not corrected for doubling artifacts (27), since the core assumption of that correction is being tested.

### Flow cytometry experiments

Strains were grown overnight in LB in deep 96-well plates, and then back-diluted 1:300 into fresh LB containing 1% xylose to induce expression of dCas9 and moved to either 30°C, 37°C, or 45°C. After ~5 doublings, cells were diluted 1:300 into phosphate-buffered saline, and green fluorescence levels (533/30 filter, 488 nm laser) were determined using a BD Accuri Flow Cytometer (BD Biosciences) as previously described (18). Data for at least 10,000 cells were collected. Data were analyzed with the R package FlowCore (28).

## Data Availability

Raw sequencing data are deposited on NCBI-SRA Bioproject PRJNA1375834.

## SUPPLEMENTAL MATERIAL

Supplemental material msphere.00379-26-s0001.docx Figures S1 to S3; caption for Table S1.

Table S1 msphere.00379-26-s0002.xlsx RF values for all sgRNAs in different media and temperatures.

ASM does not own the copyrights to Supplemental Material that may be linked to, or accessed through, an article. The authors have granted ASM a non-exclusive, world-wide license to publish the Supplemental Material files. Please contact the corresponding author directly for reuse.
