# Supplementary material for: Elevated temperature undermines the efficacy of Streptococcus pyogenes dCas9-based CRISPRi systems
Source: mSphere. 2026 Aug 19;11(9):e00379-26. doi: 10.1128/msphere.00379-26 (PMC13621834; doi:10.1128/msphere.00379-26)
Supplement: Supplementary file 1 [file msphere.00379-26-s0001.docx]

**SUPPLEMENTARY FIGURES**

**Fig. S1.** Barplot of doubling time (in minutes) for our CRISPRi library growing in 3 different media types (LB; minimal media - MM; and glucose complete - GC) at 3 different temperatures. In all comparisons growth rates were highest at 45°C, and progressively slower at 37°C and 30°C. Overall, growth rates were lowest in MM and highest in LB, reflecting the relative nutrient richness of different media.

**Fig. S2.** Boxplot of RF values for fully complementary sgRNAs targeting nonessential genes in *B. subtilis* grown in different media (LB; minimal media-MM; and glucose complete-GC) and temperature (30°C, 37°C, 45°C) conditions. High temperature is highlighted in red, low temperature is highlighted in blue. Median RF for non-essential genes was not affected by media or temperature.

**Fig. S3.** Scatterplots of RF values for fully complementary sgRNAs targeting all genes in *B. subtilis* grown in different media (LB; minimal media-MM; and glucose complete-GC) and temperature (30°C, 37°C, 45°C) conditions, comparing RFs at different temperatures within the same media. Points representing sgRNAs targeting essential genes are circled. Points are colored by functional category of gene targeted, as determined by first listed SubtiWiki [(26)](https://paperpile.com/c/TfEaSo/XBr6) category: Red - Cell envelope (SubtiWiki category 1.1), Blue - Metabolism (2), Green - DNA processes (3.1), Purple - RNA processes (3.2), Orange - Translation (3.3), Brown - Stress response (4.3). For the 37°C to 45°C comparisons, all sgRNAs with an ΔRF_37°C - 45°C_ > 0.2 (i.e., strains growing slower at 45°C) are labeled with the targeted gene name. For the 37°C to 30°C comparisons, all sgRNAs with an |ΔRF_37°C - 45°C_| > 0.2 (i.e., strains growing slower or faster at 30°C) are labeled with the targeted gene name.

**SUPPLEMENTARY TABLES**

**Table S1:** RF values for all sgRNAs in different media and temperatures.
